# Effects of Cardiac Rehabilitation and Diet Counselling on Adherence to the Mediterranean Lifestyle in Patients after Myocardial Infarction

**DOI:** 10.3390/nu14194048

**Published:** 2022-09-29

**Authors:** Marko Novaković, Uroš Rajkovič, Daniel Košuta, Jure Tršan, Zlatko Fras, Borut Jug

**Affiliations:** 1 Department of Vascular Diseases, University Medical Centre Ljubljana, Zaloška 7/VI, 1000 Ljubljana, Slovenia; 2 Faculty of Medicine, University of Ljubljana, Vrazov trg 2, 1000 Ljubljana, Slovenia; 3 Faculty of Organizational Sciences, University of Maribor, Kidričeva cesta 55a, 4000 Kranj, Slovenia

**Keywords:** Mediterranean diet, Mediterranean lifestyle, myocardial infarction, coronary artery disease, cardiac rehabilitation, lipid status, physical activity

## Abstract

Adherence to the Mediterranean lifestyle—as captured by the Medlife Index Questionnaire (i.e., encompassing a Mediterranean diet as well as other aspects of healthy living, such as food preparation, physical activity, and socializing)—has been associated with reduced cardiovascular events in healthy individuals. In the present study, we sought to determine the adherence to, and the effect of comprehensive cardiac rehabilitation on, Mediterranean lifestyle adherence in patients after myocardial infarction. We included 121 patients (mean age, 55 years; women, 37%) undergoing comprehensive cardiac rehabilitation—i.e., exercise training 3 times per week for 12 weeks plus dedicated workshops promoting the Mediterranean lifestyle. Before and after cardiac rehabilitation, patients completed the Medlife Index Questionnaire. High baseline adherence was associated with favourable glucose (5.39 vs. 6.1 mmol/L; *p* < 0.001), triglycerides (1.1 vs. 1.5 mmol/L; *p* = 0.002), and HDL cholesterol levels (1.32 vs. 1.12 mmol/L; *p* = 0.032). More importantly, the Medlife Score significantly improved following comprehensive cardiac rehabilitation in patients with low baseline adherence (from 13.8 to 16.7 points; *p* < 0.001), but not in patients with high baseline adherence (from 19.4 to 18.8 points; *p* = 0.205). Our findings suggested that Mediterranean lifestyle promotion during cardiac rehabilitation improved adherence to the Mediterranean lifestyle, especially in low-adherence patients.

## 1. Introduction

Myocardial infarction—a manifestation of coronary atherosclerotic vascular disease—remains a major cause of morbidity and mortality worldwide [1]. The management of myocardial infarction includes immediate revascularisation and the long-term secondary prevention of atherosclerotic vascular disease in the form of prophylactic medication and lifestyle intervention—i.e., smoking cessation, regular exercise, and a Mediterranean-type diet [2]. The Mediterranean diet is entangled with the way of life in Mediterranean countries [3,4,5] and is associated with favourable glucose and lipid metabolism (i.e., lower fasting plasma glucose levels, lower low-density lipoprotein (LDL) cholesterol and triglycerides levels, and low high-density (HDL) cholesterol levels) [6], improved vascular function [6], and reduced cardiovascular disease morbidity and mortality [7,8,9,10]. Pertinent guidelines for cardiovascular disease prevention, therefore, recommend Mediterranean diet for all patients after myocardial infarction [2], as it was shown to be effective for both the primary and secondary prevention of cardiovascular diseases [7,11,12,13], even as a modifier of genetic risk for cardiovascular disease [14].

Lifestyle in the Mediterranean basin, however, encompasses concepts above and beyond diet, such as food preparation, physical activity and sedentary lifestyle avoidance, and socializing, which are considered proxy for a healthy way of living [4,15]. These concepts, together with the Mediterranean diet, are captured by the Mediterranean lifestyle (Medlife) Index [16]. The Medlife Index is a recently designed questionnaire that summarises all aspects of the Mediterranean lifestyle and consists of 28 questions in three domains—food consumption, dietary habits, and social habits. So far, it has been applied in various populations in both Mediterranean and non-Mediterranean communities [16,17,18,19].

Adherence to the Mediterranean lifestyle, as captured by high scores on the Medlife Index Questionnaire, has been associated with decreased all-cause mortality in healthy individuals [17]. Conversely, data on adherence to the Mediterranean lifestyle in patients after myocardial infarction are scarce. Furthermore, as per guideline recommendations [2], patients after myocardial infarction should undergo comprehensive cardiac rehabilitation, which includes dietary counselling, behavioural support, and exercise; the effects of such programmes on adherence to the Mediterranean lifestyle, however, remain unknown. Therefore, we aimed to (i) assess adherence to the Mediterranean lifestyle in patients after myocardial infarction, (ii) appraise the determinants of adherence to the Mediterranean lifestyle, and (iii) evaluate the effects of a comprehensive cardiac rehabilitation programme on adherence to the Mediterranean lifestyle.

## 2. Materials and Methods

### 2.1. Study Population and Design

We included patients after recent myocardial infarction (<4 months prior to the study commencement), who were referred for cardiac rehabilitation programme at Centre for Preventive Cardiology, Department of Vascular Diseases, University Medical Centre Ljubljana, Slovenia. Exclusion criteria were recent acute (<1 month) or uncontrolled non-cardiovascular diseases requiring hospital admission, emergency, or unplanned specialist management; unstable dysrhythmias; pregnancy; and intellectual development disorder. The study was approved by the National Medical Ethics Committee and was performed in accordance with the Declaration of Helsinki and its later amendments. Written consent was obtained from all patients prior to their inclusion.

Before and after the intervention period (i.e., 12 weeks), blood samples were collected after at least 8 h fasting from all the patients and were analysed for blood glucose and lipid levels (direct measurement of total cholesterol, LDL cholesterol, HDL cholesterol, and triglycerides levels at the national referral Laboratory for Haemostasis and Atherothrombosis of Department of Vascular Diseases, University Medical Centre Ljubljana). At baseline, demographic characteristics were captured, including educational status (with higher education defined as the completion of >12 years of education) and living place (defined as either urban/suburban or rural as per the national statistics office definition); a thorough medical history and clinical assessment was performed, with the emphasis of cardiovascular appraisal and risk factors. Height and weight measurements were measured using a standard methodology [20]. Data on medication use were based on self-reports and checked in pharmaceutical records as needed (e.g., if a patient could not recall medication use details).

### 2.2. Medlife Index Questionnaire

The Medlife Index Questionnaire consists of 28 questions divided in three domains: food consumption, dietary habits, and social habits. Adherence to a specific item is scored with 0 or 1. The overall Medlife Index Score, therefore, comprises values between 0 and 28. According to the overall Medlife Index Score, the patient cohort was divided into tertiles [21,22] and was afterward classified into two groups: high-adherence to Medlife (the highest tertile) and low-adherence to Medlife (the lowest and medium tertiles).

All included patients filled out the Medlife questionnaire twice, at baseline and after the completion of the cardiac rehabilitation programme.

### 2.3. Cardiac Rehabilitation and Dietary Counselling

Cardiac rehabilitation is a comprehensive programme for patients after myocardial infarction. In Slovenia, it is mostly carried out at outpatient clinics and is primarily exercise-based.

This study was carried out at Centre for Preventive Cardiology, Department of Vascular Diseases, University Medical Centre Ljubljana, Slovenia, and lasted 12 weeks for each patient. Besides exercise, patients were included in (i) workshops on general health, risk factors, and cardioprotective therapy (one 90 min mandatory workshop plus as many as needed), (ii) smoking cessation for smokers, (iii) psychological assessment (with counselling on relaxation techniques and stress management as needed), (iv) and dietary counselling (one major 90 min workshop with regular (10–15 min once per week) reassessment and reinforcement during each rehabilitation session). Dietary counselling was provided by an experienced nutrition professional (specialised RN with nutrition counselling expertise). According to guidelines [2], the Mediterranean diet was promoted as the healthiest type, and the following specific suggestions were given: (i) to use olive oil as the main fat for cooking and dressing, (ii) to have at least 2 seasonal, fresh vegetable servings a day (e.g., tomato, broccoli, spinach, pepper, eggplant, cabbage, lettuce, zucchini, etc.), (iii) moderate consumption of fresh fruit (e.g., oranges, tangerines, apples, berries, pears, grapes, peaches, apricots, nectarines, figs, melons), (iv) consumption of fish at least 2 times per week, (v) favouring legumes (e.g., beans, peas, lentils, chickpeas, soybean) to meat (especially red meat), and (vi) having a handful of unsalted nuts every day (e.g., walnuts, hazelnuts, peanuts, almonds, cashews, macadamia nuts). A serving was defined as approximately 1 cup = 250 mL. In addition, advice regarding food preparation was given, e.g., favouring cooking to frying and limiting the use of salt and sugar. Finally, a few examples of healthy meals (including information on ingredients and recipes) were provided.

During the workshops on general health, risk factors, and cardioprotective therapy, patients were advised to have enough sleep during the night and to minimise sedentary lifestyle (e.g., watching television or using the computer) and were encouraged to enjoy outdoor activities (e.g., light gardening).

Despite recommendations on Mediterranean lifestyle regarding exercise being carried out outdoor, due to safety reasons (patients shortly after myocardial infarction), exercise training sessions were held 3 times per week for one hour in the morning hours indoor; exercise training sessions consisted of supervised aerobic exercise and training exercise on exercise bike or treadmill with adjunction of low-weight resistance training.

### 2.4. Statistical Analysis

The normal distribution of variables was determined using the Shapiro–Wilk test. Normally distributed variables were described with a mean value and standard deviation. Asymmetrically distributed variables were described with medians and interquartile ranges. Categorical variables were described as numbers and/or percentages. Differences between two groups were determined with the independent samples *t*-test and Mann–Whitney U test for normally and asymmetrically distributed variables, respectively. Differences in categorical variables (percentages) were assessed with the chi-squared test. The effects of the intervention were determined with the paired samples *t*-test and Wilcoxon U paired test for normally and asymmetrically distributed variables, respectively. The analysis of covariance (ANCOVA) test was performed to assess the post-rehabilitation Medlife Index Score with respect to baseline pre-rehabilitation values. All data were analysed using IBM SPSS Statistics v. 20 (IBM Corp., Armonk, NY, USA). A *p*-value < 0.05 was considered statistically significant.

## 3. Results

We included 121 patients with a mean age of 55 years, and 33 (27%) were women. Based on the adherence to the Mediterranean lifestyle, patients were divided into high-adherence (upper Medlife Index tertile) and low-adherence (first and second Medlife Index tertiles) groups.

There were no significant differences between groups in terms of age, sex, BMI, clinical data, drug prescription, and risk factors (arterial hypertension, dyslipidaemia, cigarette smoking, and diabetes mellitus). In addition, there were no significant differences in terms of living place (Table 1). Conversely, patients in the high-adherence group had significantly more prevalent family history of CVD (55.9% vs. 34.5%; *p* = 0.031) and a larger percentage of higher educated people (47.1% vs. 26.4%; *p* = 0.029) than the low-adherence group.

At baseline, patients in the high-adherence group had significantly lower blood glucose (5.39 vs. 6.08 mmol/L; *p* = 0.001), HbA1C (5.60% vs. 5.89%; *p* = 0.027), and triglyceride levels (1.17 vs. 1.54 mmol/L; *p* = 0.002) than patients in the low-adherence group. These differences mostly remained significant at follow-up, with 5.41 vs. 6.12 mmol/L (*p* < 0.001) for blood glucose levels, 1.32 vs. 1.14 mmol/L (*p* = 0.007) for HDL, and 1.05 vs. 1.70 mmol/L (*p* < 0.001) for triglyceride levels, when high- and low-adherence groups were compared, respectively (Table 2).

Cardiac rehabilitation and diet counselling were not associated with a significant post-intervention change in the lipid status in either of the groups (Table 2), except for a significant increase in HDL levels in the high-adherence group (from 1.26 to 1.32 mmol/L; *p* = 0.045).

**Figure 1:**
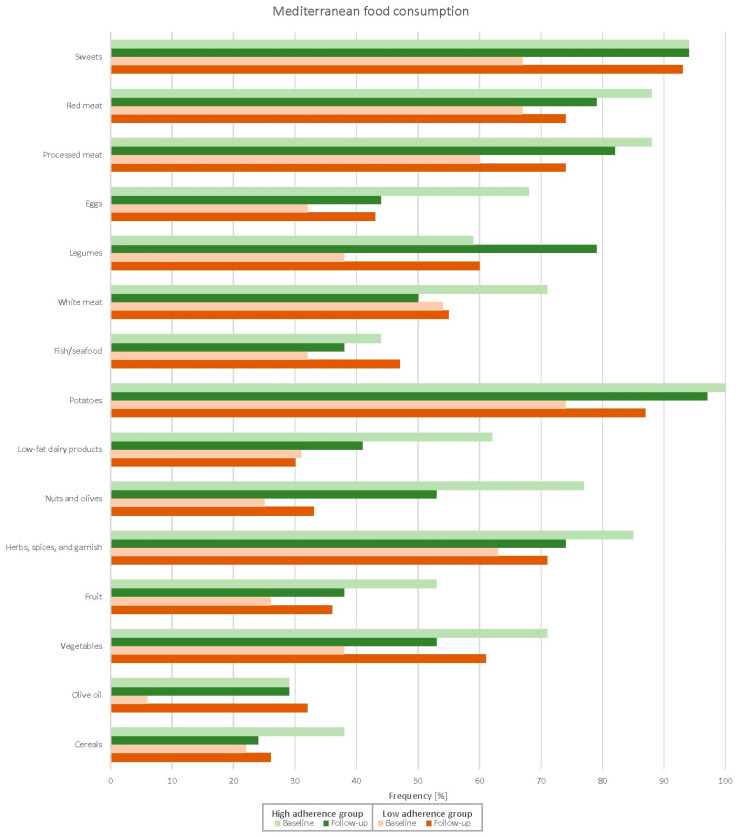
Changes in the Mediterranean food consumption subdomains after the cardiac rehabilitation programme.

**Figure 2:**
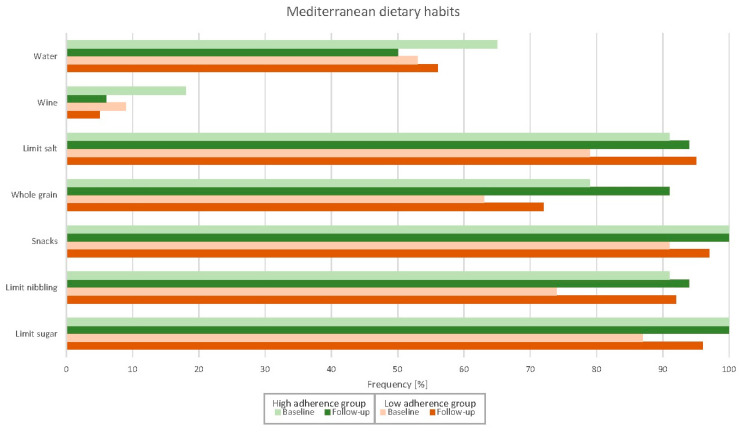
Changes in the Mediterranean dietary subdomains after the cardiac rehabilitation programme.

**Figure 3:**
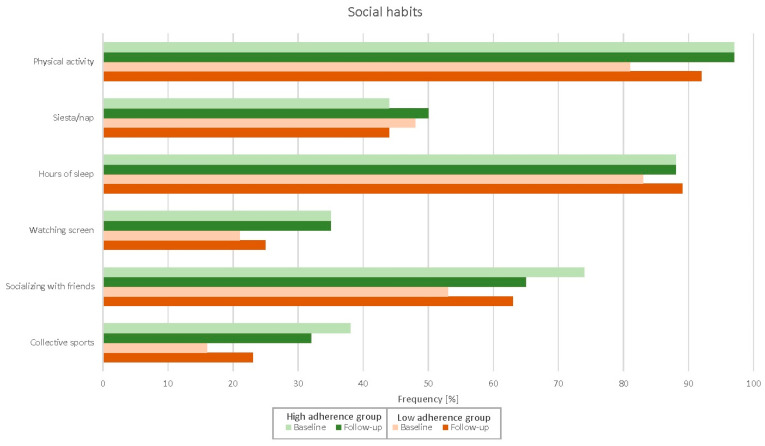
Changes in the social habits subdomains after the cardiac rehabilitation programme.

Figure 1, Figure 2 and Figure 3 and Table A1 depict adherence to the specific domains in the Medlife questionnaire of both high- and low-adherence groups at baseline and follow-up. Patients in the high-adherence group were more adherent to most Mediterranean food consumption domains than the low-adherence group. Patients in the low-adherence group significantly improved adherence in several domains, while patients in the high-adherence did not. Similarly, the Medlife Index Score significantly improved only in the low-adherence group (from 13.8 to 16.7 points; *p* < 0.001), while in the high-adherence group, it did not (from 19.4 to 18.8 points; *p* = 0.205).

Table 3 shows the ANCOVA tests using follow-up overall Medlife Index Score (a) and overall Medlife Index Score difference (b) as dependent variables, high vs. low adherence as a fixed factor, and baseline overall Medlife Index Score as a covariate in both models. The covariate, i.e., the baseline overall Medlife Index Score, was significantly related to both dependent variables. There was also a significant effect of the high- vs. low-adherence groups on both dependent variables (Table 3).

## 4. Discussion

Our study showed that adherence to the Mediterranean lifestyle was associated with more favourable glucometabolic and lipid profiles in patients after myocardial infarction. More importantly, our findings suggest that poor adherence to the Mediterranean lifestyle—as captured by a low score on the Medlife Index Questionnaire—may be improved by a comprehensive cardiac rehabilitation programme, which provides dietary counselling as part of a multifaceted lifestyle intervention through cardiac rehabilitation. To the best of our knowledge, this is the first study in patients after myocardial infarction to assess the adherence to, and improvement of, a Mediterranean lifestyle, which encompasses diverse aspects of living beyond and above food consumption (i.e., food preparing, physical activity, and socializing).

Patients who adhered to the Mediterranean lifestyle after myocardial infarction had better glucose and lipid profiles. In our study, high adherence (i.e., highest-tertile Medlife Index Score) was associated with lower levels of fasting blood glucose, as well as lower triglycerides and higher HDL-C levels, which corroborated previous findings in healthy individuals [6,18,23]. Conversely, in contrast to previous studies in healthy individuals [24], we found no associations with LDL-C levels. This is in line with our present understanding of derangements in lipid metabolism. Unfavourable triglyceride/HDL cholesterol levels are associated with obesity, diabetes, unhealthy dietary habits, and sedentary lifestyle; are predictors of atherosclerotic vascular disease onset and progression (“atherogenic dyslipidaemia”); and—as opposed to LDL cholesterol—are less amendable via lipid-lowering medication [25] but responsive to lifestyle interventions [26,27,28]. In our study, all patients were on guideline-directed high-intensive statin therapy, which likely overshadowed the effect of lifestyle on LDL cholesterol; conversely, favourable triglyceride/HDL cholesterol levels were strongly associated with adherence to the Mediterranean lifestyle, underscoring the importance of lifestyle in addressing residual cardiovascular risk, which is not amendable via lipid-lowering medication.

More importantly, adherence to the Mediterranean lifestyle significantly improved after a comprehensive cardiac rehabilitation programme—but only in patients with a lower baseline Medlife Index Score. The difference between the low- and high-adherence groups diminished to non-significant levels after cardiac rehabilitation, except for legume, vegetable, nut, olive, olive oil, and whole-grain product consumption; this represents the largest room for improvement in patients with poor baseline adherence. While intuitive in terms of a “regression to the mean” phenomenon, our findings remained significant after ANCOVA adjustment for baseline Medlife Index Scores. Moreover, some aspects of the Mediterranean lifestyle—such as legume and whole-grain product consumption—improved even in patients with high baseline adherence. Conversely, patients with low baseline adherence improved in most Mediterranean diet and dietary pattern domains. Altogether, these findings suggest that patients with low adherence to the Mediterranean lifestyle, as appraised with the Medlife Index Questionnaire, may benefit the most from lifestyle interventions. In addition, there is room for improvement even in patients with high baseline adherence—especially in below-satisfactory domains, such as the consumption of nuts and olives (53%), vegetables (53%), and olive oil (29%).

Two findings in our study merit further addressing. Firstly, family history of cardiovascular disease was associated with higher adherence to the Mediterranean lifestyle. It seems that experiencing a cardiovascular event in the immediate family may impact lifestyle choices—either through alarming (i.e., offspring of parents with cardiovascular disease trying to defer atherosclerotic events) or empowerment (i.e., vertical sharing and spreading the Mediterranean lifestyle from parents’ secondary prevention experience). However intriguing, these speculations require further appraisal with properly designed studies. Secondly, sociodemographic factors were important determinants of Mediterranean lifestyle adherence. Education level was associated with a higher Medlife Index Scores in our study—a finding comparable to some [29,30]—but not all [18]—reports on healthy individuals. Lower household income [31] and socioeconomic status [32] were also previously associated with poor adherence, whereas our study did not show a possible impact of living place (urban/suburban vs. rural). While most reports in healthy individuals suggested a better adherence to the Mediterranean diet in rural communities [33], the uptake of other Mediterranean lifestyle domains, such as regular exercise, may be less pronounced [29,30]. The promotion of the Mediterranean lifestyle, therefore, should be applied even to communities with a perceived Mediterranean way of life, which may be prone to a “westernization” or “nutrition transition” of lifestyle patterns [34,35].

We identified some limitations of our study—firstly, sample size, design, and the single-centre setting. Its quasi-experimental pre–post-test series design limited the data quality and the evidence that could be derived from them. Although the sample size in this report was fairly large as compared with similar prospective cohorts, larger and randomised, controlled, multicentre studies are warranted to confirm and further explicate our results. Secondly, the extrapolation of our results to populations beyond coronary artery disease patients, especially in terms of lipid status, should be performed with caution, as all our patients after myocardial infarction exhibited lipid profiles compatible with high-intensity statin management. Thirdly, using categorical variables—not continuous scale values—was already identified as a limitation of the original study that introduced the Medlife Index Questionnaire [10]. Fourthly, data were obtained from the questionnaire, so imprecision and the response and social desirability biases could not be ruled-out. Finally, even at follow-up, a large percentage of patients did not adopt the classical recommendations of the Mediterranean lifestyle (e.g., olive oil, vegetables, or nuts) suggesting that the implementation strategies for improvement should be refined.

## 5. Conclusions

In conclusion, we showed that baseline adherence to the Mediterranean lifestyle was associated with more favourable glucose and lipid profiles in patients after myocardial infarction. More importantly, a comprehensive cardiac rehabilitation programme, which included Mediterranean lifestyle promotion, effectively improved adherence to the Mediterranean lifestyle—especially in low-adherence patients—which was a main strength of our report. The Mediterranean lifestyle in patients after myocardial infarction is a pivotal guideline-derived intervention, which is associated with a favourable cardiometabolic profile and should be especially encouraged in patients with poor baseline adherence.

## Figures and Tables

**Table 1:** Baseline demographics and clinical data.

	All (n = 121)	Low Adherence (n = 87)	High Adherence (n = 34)	Significance
*Demographic*				
Age, mean (SD), years	56.0 (9.7)	56.4 (9.1)	54.9 (11.0)	0.501
Female sex, n (%)	33 (27.3%)	23 (26.4%)	10 (29.4)	0.741
Height, mean (SD), cm	173.0 (8.8)	172.7 (8.3)	173.6 (10.0)	0.655
Weight, mean (SD), kg	85.9 (17.0)	86.6 (17.5)	84.2 (15.7)	0.464
BMI, mean (SD), kg/m^2^	28.6 (4.5)	28.9 (4.7)	27.8 (4.0)	0.221
Normal weight, n (%)	24 (19.8%)	16 (18.4%)	8 (23.5%)	0.524
Overweight, n (%)	58 (47.9%)	40 (46.0%)	18 (53.0%)	0.491
Obese, n (%)	39 (32.2%)	31 (35.6%)	8 (23.5%)	0.200
*Risk factor for CVD*				
Arterial hypertension, n (%)	55 (45.5%)	38 (43.7%)	17 (50.0%)	0.530
Dyslipidaemia, n (%)	70 (57.9%)	54 (62.1%)	16 (47.1%)	0.133
Family history of CVD, n (%)	49 (40.5%)	30 (34.5%)	19 (55.9%)	0.031
Diabetes mellitus, n (%)	13 (10.7%)	10 (11.5%)	3 (8.8%)	0.670
Current cigarette smoker, n (%)	16 (13.2%)	13 (15.0%)	3 (8.8%)	0.216
Former cigarette smoker, n (%)	42 (34.7%)	33 (37.9%)	9 (26.5%)	
Never smoked, n (%)	63 (52.1%)	41 (47.1%)	22 (64.7%)	
*Clinical data*				
STEMI, n (%)	75 (62.0%)	54 (62.1%)	21 (61.7%)	0.975
Ejection fraction, median (Q1–Q3), %	55 (53–56)	55 (54–56)	55 (50–59)	0.998
PCI LAD, n (%)	65 (53.7%)	49 (56.3%)	16 (47.1%)	0.358
*Medication*				
ACEi/ARB, n (%)	100 (82.6%)	72 (82.8%)	28 (82.3%)	0.958
Beta blockers, n (%)	105 (86.8%)	76 (87.4%)	29 (85.3%)	0.763
Statins, n (%)	121 (100%)	87 (100%)	34 (100%)	1.000
Antidiabetic therapy, n (%)	10 (8.3%)	8 (9.2%)	2 (5.9%)	0.552
*Laboratory test*				
HbA1c, mean (SD), %	5.81 (0.75)	5.89 (0.82)	5.60 (0.48)	0.027
*Educational level*				
Lower, n (%)	82 (67.8%)	64 (73.6%)	18 (52.9%)	0.029
Higher, n (%)	39 (32.2%)	23 (26.4%)	16 (47.1%)	
*Living place*				
Urban and suburban, n (%)	89 (45.4%)	62 (40.2%)	27 (58.8%)	0.361
Rural, n (%)	32 (26.4%)	25 (28.7%)	7 (20.6%)	

SD—standard deviation; BMI—body mass index; CVD—cardiovascular disease; STEMI—ST-elevation myocardial infarction; Q1–Q3—interquartile range; PCI LAD—percutaneous coronary intervention of left anterior descending artery; ACEi/ARB—angiotensin-converting enzyme inhibitor/angiotensin receptor blocker; HbA1c—glycated haemoglobin.

**Table 2:** Effects of cardiac rehabilitation and diet counselling on blood glucose and lipid status in both examined groups.

	Low Medlife Adherence (n = 87)			High Medlife Adherence (n = 34)			Comparison between Groups (*p*)	
	Baseline	Follow-Up	*p*	Baseline	Follow-Up	*p*	Baseline	Follow-Up
Glucose	6.08 (1.47)	6.12 (1.43)	0.569	5.39 (0.63)	5.41 (0.71)	0.903	0.001	<0.001
TChol	3.44 (0.87)	3.45 (0.91)	0.928	3.32 (0.83)	3.10 (0.67)	0.236	0.505	0.034
HDL	1.12 (0.33)	1.14 (0.36)	0.630	1.26 (0.35)	1.32 (0.34)	0.045	0.032	0.007
LDL	1.61 (0.70)	1.57 (0.66)	0.431	1.54 (0.74)	1.34 (0.60)	0.272	0.398	0.068
Triglycerides	1.54 (0.71)	1.70 (1.28)	0.313	1.17 (0.63)	1.05 (0.37)	0.338	0.002	<0.001

All measurements are described as mean (SD) and expressed in mmol/L. TChol—total cholesterol; HDL-c—high-density lipoprotein cholesterol; LDL-c—low-density lipoprotein cholesterol.

**Table 3:** ANCOVA tests using high vs. low adherence as a fixed factor, baseline overall Medlife Index score as a covariate, and (a) follow-up overall Medlife Index score and (b) overall Medlife Index score difference, as dependent variables.

(a) Dependent Variable: Follow-Up Overall Medlife Index Score						
**Source**	Type III Sum of Squares	*df*	Mean Square	F	Sig.	Partial Eta Squared
Corrected model	347.806 *	2	173.903	30.255	0.000	0.339
Intercept	69.921	1	69.921	12.164	0.001	0.093
Baseline overall Medlife Index Score	240.060	1	240.060	41.764	0.000	0.261
High adherence vs. Low adherence	31.369	1	31.369	5.457	0.021	0.044
Error	678.260	118	5.748			
Total	37,299.000	121				
Corrected total	1026.066	120				
**(b) Dependent Variable: Overall Medlife Index Score Difference**						
**Source**	**Type III Sum of Squares**	***df***	**Mean Square**	**F**	**Sig.**	**Partial Eta Squared**
Corrected model	355.211 **	2	177.605	30.899	0.000	0.344
Intercept	69.921	1	69.921	12.164	0.001	0.093
Baseline overall Medlife Index Score	47.355	1	47.355	8.239	0.005	0.065
High adherence vs. Low adherence	31.369	1	31.369	5.457	0.021	0.044
Error	678.260	118	5.748			
Total	1486.000	121				
Corrected total	1033.471	120				

Legend: * R squared = 0.339 (adjusted R squared = 0.328), ** R squared = 0.344 (adjusted R squared = 0.333).

## Data Availability

Data are available from the corresponding author upon reasonable request.

## Previous Presentations

Parts of these results were presented as an abstract at the European Society of Cardiology-Preventive Cardiology Congress 2021 (abstract 11491).

## Appendix A

**Table A1:** Changes in the specific domains of the Medlife questionnaire after the cardiac rehabilitation pro-gramme and comparison between groups.

	High Adherence to Mediterranean Lifestyle (n = 34)			Low Adherence to Mediterranean Lifestyle (n = 87)			Comparison between Groups	
	Baseline	Follow-Up	*p*	Baseline	Follow-Up	*p*	Baseline	Follow-up
Medlife index	19.4 (1.8)	18.8 (3.0)	0.205	13.8 (2.2)	16.7 (2.7)	<0.001	<0.001	0.001
Mediterranean food consumption								
Sweets (≤2 servings/week)	94%	94%	1.000	67%	93%	<0.001	0.004	1.000
Red meat (<2 servings/week)	88%	79%	0.257	67%	74%	0.083	0.030	0.663
Processed meat (≤1 serving/week)	88%	82%	0.414	60%	74%	0.014	0.005	0.435
Eggs (2–4 servings/week)	68%	44%	0.033	32%	43%	0.061	0.001	1.000
Legumes (≥2 servings/week)	59%	79%	0.008	38%	60%	<0.001	0.060	0.068
White meat (2 servings/week)	71%	50%	0.090	54%	55%	0.847	0.145	0.756
Fish/seafood (≥2 servings/week)	44%	38%	0.414	32%	47%	0.007	0.307	0.496
Potatoes (≤3 servings/week)	100%	97%	0.317	74%	87%	0.003	0.002	0.175
Low-fat dairy products (2 servings/day)	62%	41%	0.071	31%	30%	0.841	0.004	0.331
Nuts and olives (1–2 servings/day)	77%	53%	0.021	25%	33%	0.194	<0.001	0.075
Herbs, spices, and garnish (≥1 servings/day)	85%	74%	0.102	63%	71%	0.144	0.032	0.981
Fruit (3–6 servings/day)	53%	38%	0.197	26%	36%	0.170	0.011	0.954
Vegetables (≥2 servings/day)	71%	53%	0.058	38%	61%	<0.001	0.002	0.551
Olive oil (≥3 servings/day)	29%	29%	1.000	6%	32%	<0.001	0.001	0.938
Cereals (3–6 servings/day)	38%	24%	0.132	22%	26%	0.414	0.108	0.922
Mediterranean dietary habits								
Water or infusions	65%	50%	0.197	53%	56%	0.480	0.329	0.589
Wine	18%	6%	0.102	9%	5%	0.206	0.214	0.673
Limit salt in meals	91%	94%	0.317	79%	95%	<0.001	0.200	0.673
Preference for whole-grain products	79%	91%	0.046	63%	72%	0.059	0.134	0.047
Snacks	100%	100%	1.000	91%	97%	0.096	0.104	0.558
Limit nibbling between meals	91%	94%	0.564	74%	92%	<0.001	0.061	1.000
Limit sugar in beverages	100%	100%	1.000	87%	96%	0.005	0.033	0.558
Social habits								
Physical activity 150 min/week	97%	97%	1.000	81%	92%	0.012	0.043	0.439
Siesta/nap	44%	50%	0.480	48%	44%	0.394	0.834	0.671
Hours of sleep	88%	88%	1.000	83%	89%	0.166	0.641	1.000
Watching television	35%	35%	1.000	21%	25%	0.248	0.150	0.381
Socializing with friends	74%	65%	0.317	53%	63%	0.050	0.062	1.000
Collective sports	38%	32%	0.480	16%	23%	0.134	0.017	0.437

## References

[1] Reed G.W., Rossi J.E., Cannon C.P. (2017). Acute Myocardial Infarction. Lancet.

[2] Visseren F.L.J., Mach F., Smulders Y.M., Carballo D., Koskinas K.C., Bäck M., Benetos A., Biffi A., Boavida J.-M., Capodanno D. (2021). 2021 ESC Guidelines on Cardiovascular Disease Prevention in Clinical Practice. Eur. Heart J..

[3] Finicelli M., Di Salle A., Galderisi U., Peluso G. (2022). The Mediterranean Diet: An Update of the Clinical Trials. Nutrients.

[4] Mazzocchi A., Leone L., Agostoni C., Pali-Schöll I. (2019). The Secrets of the Mediterranean Diet. Does [Only] Olive Oil Matter?. Nutrients.

[5] La Torre G., Saulle R., Di Murro F., Siliquini R., Firenze A., Maurici M., Mannocci A., Colamesta V., Barillà F., Ferrante F. (2018). Mediterranean Diet Adherence and Synergy with Acute Myocardial Infarction and Its Determinants: A Multicenter Case-Control Study in Italy. PLoS ONE.

[6] Esposito K., Marfella R., Ciotola M., Di Palo C., Giugliano F., Giugliano G., D’Armiento M., D’Andrea F., Giugliano D. (2004). Effect of a Mediterranean-Style Diet on Endothelial Dysfunction and Markers of Vascular Inflammation in the Metabolic Syndrome: A Randomized Trial. JAMA.

[7] Delgado-Lista J., Alcala-Diaz J.F., Torres-Peña J.D., Quintana-Navarro G.M., Fuentes F., Garcia-Rios A., Ortiz-Morales A.M., Gonzalez-Requero A.I., Perez-Caballero A.I., Yubero-Serrano E.M. (2022). Long-Term Secondary Prevention of Cardiovascular Disease with a Mediterranean Diet and a Low-Fat Diet (CORDIOPREV): A Randomised Controlled Trial. Lancet.

[8] Eguaras S., Toledo E., Hernández-Hernández A., Cervantes S., Martínez-González M. (2015). Better Adherence to the Mediterranean Diet Could Mitigate the Adverse Consequences of Obesity on Cardiovascular Disease: The SUN Prospective Cohort. Nutrients.

[9] Booth J.N., Levitan E.B., Brown T.M., Farkouh M.E., Safford M.M., Muntner P. (2014). Effect of Sustaining Lifestyle Modifications (Nonsmoking, Weight Reduction, Physical Activity, and Mediterranean Diet) After Healing of Myocardial Infarction, Percutaneous Intervention, or Coronary Bypass (from the REasons for Geographic and Racial Differences in Stroke Study). Am. J. Cardiol..

[10] Shikany J.M., Safford M.M., Bryan J., Newby P.K., Richman J.S., Durant R.W., Brown T.M., Judd S.E. (2018). Dietary Patterns and Mediterranean Diet Score and Hazard of Recurrent Coronary Heart Disease Events and All-Cause Mortality in the REGARDS Study. JAHA.

[11] Estruch R., Ros E., Salas-Salvadó J., Covas M.-I., Corella D., Arós F., Gómez-Gracia E., Ruiz-Gutiérrez V., Fiol M., Lapetra J. (2018). Primary Prevention of Cardiovascular Disease with a Mediterranean Diet Supplemented with Extra-Virgin Olive Oil or Nuts. N. Engl. J. Med..

[12] Liang K.-W., Lee C.-L., Liu W.-J. (2022). Lower All-Cause Mortality for Coronary Heart or Stroke Patients Who Adhere Better to Mediterranean Diet-An NHANES Analysis. Nutrients.

[13] Vázquez-Ruiz Z., Toledo E., Vitelli-Storelli F., Goni L., de la O V., Bes-Rastrollo M., Martínez-González M.Á. (2022). Effect of Dietary Phenolic Compounds on Incidence of Cardiovascular Disease in the SUN Project; 10 Years of Follow-Up. Antioxidants.

[14] Leu H.-B., Chung C.-M., Chen J.-W., Pan W.-H. (2019). The Mediterranean Diet Reduces the Genetic Risk of Chromosome 9p21 for Myocardial Infarction in an Asian Population Community Cohort. Sci. Rep..

[15] Noites A., Pinto J., Freitas C.P., Melo C., Albuquerque A., Teixeira M., Bastos J.M. (2015). Effects of the Mediterranean Diet and Exercise in Subjects with Coronary Artery Disease. Rev. Port. Cardiol..

[16] Sotos-Prieto M., Moreno-Franco B., Ordovás J.M., León M., Casasnovas J.A., Peñalvo J.L. (2015). Design and Development of an Instrument to Measure Overall Lifestyle Habits for Epidemiological Research: The Mediterranean Lifestyle (MEDLIFE) Index. Public Health Nutr..

[17] Hershey M.S., Fernandez-Montero A., Sotos-Prieto M., Kales S., Gea A., Ruiz-Estigarribia L., Sánchez-Villegas A., Díaz-Gutiérrez J., Martínez-González M.A., Ruiz-Canela M. (2020). The Association Between the Mediterranean Lifestyle Index and All-Cause Mortality in the Seguimiento Universidad de Navarra Cohort. Am. J. Prev. Med..

[18] Pavičić Žeželj S., Kenđel Jovanović G., Dragaš Zubalj N., Mićović V., Sesar Ž. (2018). Associations between Adherence to the Mediterranean Diet and Lifestyle Assessed with the MEDLIFE Index among the Working Population. IJERPH.

[19] Hershey M.S., Sanchez-Villegas A., Sotos-Prieto M., Fernandez-Montero A., Pano O., Lahortiga-Ramos F., Martínez-González M.Á., Ruiz-Canela M. (2022). The Mediterranean Lifestyle and the Risk of Depression in Middle-Aged Adults. J. Nutr..

[20] Olfert M., Barr M., Charlier C., Famodu O., Zhou W., Mathews A., Byrd-Bredbenner C., Colby S. (2018). Self-Reported vs. Measured Height, Weight, and BMI in Young Adults. IJERPH.

[21] Chrysohoou C., Panagiotakos D.B., Pitsavos C., Das U.N., Stefanadis C. (2004). Adherence to the Mediterranean Diet Attenuates Inflammation and Coagulation Process in Healthy Adults. J. Am. Coll. Cardiol..

[22] Hershey M.S., Sotos-Prieto M., Ruiz-Canela M., Christophi C.A., Moffatt S., Martínez-González M.Á., Kales S.N. (2021). The Mediterranean Lifestyle (MEDLIFE) Index and Metabolic Syndrome in a Non-Mediterranean Working Population. Clin. Nutr..

[23] Davis C.R., Bryan J., Hodgson J.M., Woodman R., Murphy K.J. (2017). A Mediterranean Diet Reduces F2-Isoprostanes and Triglycerides among Older Australian Men and Women after 6 Months. J. Nutr..

[24] Bédard A., Riverin M., Dodin S., Corneau L., Lemieux S. (2012). Sex Differences in the Impact of the Mediterranean Diet on Cardiovascular Risk Profile. Br. J. Nutr..

[25] Mach F., Baigent C., Catapano A.L., Koskinas K.C., Casula M., Badimon L., Chapman M.J., De Backer G.G., Delgado V., Ference B.A. (2020). 2019 ESC/EAS Guidelines for the Management of Dyslipidaemias: Lipid Modification to Reduce Cardiovascular Risk. Eur. Heart J..

[26] Jakše B., Jakše B., Pinter S., Jug B., Godnov U., Pajek J., Fidler Mis N. (2019). Dietary Intakes and Cardiovascular Health of Healthy Adults in Short-, Medium-, and Long-Term Whole-Food Plant-Based Lifestyle Program. Nutrients.

[27] Fras Z., Jug B., Penson P.E., Rizzo M. (2021). Challenges and Opportunities on Lipid Metabolism Disorders Diagnosis and Therapy: Novel Insights and Future Perspective. Metabolites.

[28] Gupta M., Jug B., Budoff M.J. (2010). Management of Cholesterol in Diabetes—A Review. US Cardiol. Rev..

[29] Kapelios C.J., Kyriazis I., Ioannidis I., Dimosthenopoulos C., Hatziagelaki E., Liatis S., The PERSEAS Study Group (2017). Diet, Life-Style and Cardiovascular Morbidity in the Rural, Free Living Population of Elafonisos Island. BMC Public Health.

[30] Kolčić I., Relja A., Gelemanović A., Miljković A., Boban K., Hayward C., Rudan I., Polašek O. (2016). Mediterranean Diet in the Southern Croatia—Does It Still Exist?. Croat. Med. J..

[31] Bonaccio M., Bonanni A.E., Di Castelnuovo A., De Lucia F., Donati M.B., de Gaetano G., Iacoviello L. (2012). On behalf of the Moli-sani Project Investigators Low Income Is Associated with Poor Adherence to a Mediterranean Diet and a Higher Prevalence of Obesity: Cross-Sectional Results from the Moli-Sani Study. BMJ Open.

[32] Mendonça N., Gregório M.J., Salvador C., Henriques A.R., Canhão H., Rodrigues A.M. (2022). Low Adherence to the Mediterranean Diet Is Associated with Poor Socioeconomic Status and Younger Age: A Cross-Sectional Analysis of the EpiDoC Cohort. Nutrients.

[33] Mattavelli E., Olmastroni E., Bonofiglio D., Catapano A.L., Baragetti A., Magni P. (2022). Adherence to the Mediterranean Diet: Impact of Geographical Location of the Observations. Nutrients.

[34] Cuschieri S., Libra M. (2020). Adherence to the Mediterranean Diet in Maltese Adults. IJERPH.

[35] Santulli G., Pascale V., Finelli R., Visco V., Giannotti R., Massari A., Morisco C., Ciccarelli M., Illario M., Iaccarino G. (2019). We Are What We Eat: Impact of Food from Short Supply Chain on Metabolic Syndrome. JCM.

